# The London Dental Hospital Students' Dinner

**Published:** 1893-12-09

**Authors:** 


### THE LONDON DENTAL HOSPITAL STUDENTS' DINNER.

It was but natural that the thoughts of the past and present students of the Dental Hospital of London, who attended the annual re-union held in the Hotel Metropole on Saturday evening, should turn upon the present position and prospects of their hospital and school. An important step is about to be taken. In order that the demands upon the charity and the school may be more adequately met the managers of the institution have resolved to build a new hospital. With this view a special effort is being made to raise £40,000. Of this a sum amounting to about £10,000, of which the members of the staff and past and present students have subscribed over £6,000, has come in. A larger sum must, however, be collected before the managers will feel justified in proceeding with their plans.

The dinner was in every respect a success. The company was large, but where sons of the same *Alma Mater* are gathered together conviviality and good fellowship are seldom wanting. They certainly were not wanting on Saturday among the past and present students of the Dental Hospital. Everybody felt that it was an honour to have the President of the Royal College of Surgeons as chairman of the gathering—the fact was alone a testimony as to the high position which dental science has now attained.

Mr. Hulke, who occupied the chair, remarked that "unless we retain the student habit to the end of our days our lot is not a very happy one." Having further illustrated from his own experience the progress which dental science has made during this generation, his sketches of the blacksmith's shop of his boyhood, which had inscribed above the door, "Tooth-drawing Bleeding and Cupping," and of the pelican, the instrument that the blacksmith used; of the two men in the market place in Auvergne who proclaimed their readiness to draw teeth and cut corns, and who did so to the music of a band that drowned all the remonstrances and cries of the victims; and of the village where he served his apprenticeship, where nobody had any idea of doing anything for teeth except pulling them out, were very graphic. But the most gratifying testimony that could have been given as to the present position of dental science, was that Mr. Hulke should express on behalf of the Royal College of Surgeons the deepest interest in the science; and he carried his audience entirely with him when he advised dental students to become qualified medical practitioners. They of the College of Surgeons, he said, were proud to have as fellows not a few who were engaged in dental practice.

Dr. Sydney Coupland, who replied for "The Visitors," made an amusing speech. Unfortunately, he said, he could only speak of dentistry as a victim, not as a practitioner. Dentistry to his thinking combined the delicacy of a fine art with the audacity of a science. How much wider was the scope of the dentist than that of the medical practitioner! Every man had thirty-two teeth but only one liver. Moreover, as civilisation advanced did not the teeth deteriorate, and there was more work for the dentist. Some people said that man would become edentulous; he did not believe that, for should man show the slightest tendency in that direction the Dental Hospital would be ready to supply him with an artificial set of teeth. Dr. Coupland's merry thrusts were received with much laughter; but, indeed, it was a merry evening throughout, and for those who had the privilege of being present it will form a pleasant memory for some time to come.

